# Pathology and Protein Changes of the Spinal Dural Arteriovenous Fistula Arterial Draining Vein Under Sustained High Vascular Pressure

**DOI:** 10.3389/fneur.2021.713355

**Published:** 2021-09-24

**Authors:** Peixi Liu, Yuan Shi, Sichen Li, Yingjun Liu, Yingjie Zhou, Yaying Song, Wei Zhu, Qingzhu An

**Affiliations:** ^1^Department of Neurosurgery, Huashan Hospital of Fudan University, Shanghai, China; ^2^Neurosurgical Institute of Fudan University, Shanghai, China; ^3^Shanghai Clinical Medical Center of Neurosurgery, Shanghai, China; ^4^Shanghai Key Laboratory of Brain Function and Restoration and Neural Regeneration, Shanghai, China; ^5^Department of Hand Surgery, Huashan Hospital, Fudan University, Shanghai, China; ^6^Department of Neurology, Renji Hospital, School of Medicine, Shanghai Jiao Tong University, Shanghai, China

**Keywords:** SDAVF draining vein, superficial temporal artery, superficial temporal vein, pathology, proteomics

## Abstract

**Object:** Spinal dural arteriovenous fistula (SDAVF) is the most common spinal vascular shunt lesion. Although pathological changes in the SDAVF draining vein (SDAVF-DV) have been elucidated, protein changes remain enigmatic. We investigated the pathology and protein changes in the SDAVF-DV under sustained high vascular pressure.

**Methods:** Three SDAVF-DV samples were compared with superficial temporal artery (STA) and superficial temporal vein (STV) samples as controls. Vascular structure was revealed by hematoxylin and eosin (H&E) and Masson staining; and cell distribution, extracellular matrix, and inflammation infiltration were observed by immunohistochemistry. Label-free quantitative proteomics was performed, and the peptide mixture was fractionated and analyzed by liquid chromatography–tandem mass spectrometry (LC-MS/MS) to identify differentially expressed proteins. Bioinformatics analysis of the differentially expressed proteins was performed using Gene Ontology (GO), Kyoto Encyclopedia of Genes and Genomes (KEGG), and protein–protein interaction (PPI) networks.

**Results:** H&E and Masson staining showed an artery-like structure of the SDAVF-DV. Immunostaining showed that vWF+ cells were not continuous in the SDAVF-DV. Although α-SMA+ and AT1+ cells were more abundant in the STV than in the SDAVF-DV, piezo-1 expression was lower in the SDAVF-DV. The SDAVF-DV showed different distributions of elastin, COL I, and COL III. COL IV and COL VI were decreased in the SDAVF-DV, while CD45+ cells and COX-1 were increased compared with those in the controls. No differences in CD68 expression and COX-2 staining were observed between the SDAVF-DV and controls. Compared with the STA, 95 proteins were upregulated and 303 proteins were downregulated in the SDAVF-DV. The most differential GO terms in each category were the adenylate cyclase-modulating G protein-coupled receptor signaling pathway, U6 snRNP, and SH3 domain binding. The most differentially expressed KEGG protein pathway was focal adhesion. Compared with the STV, the SDAVF-DV had 158 upregulated proteins and 362 downregulated proteins. The most differential GO terms in each category were lamellipodium assembly, U6 snRNP, and SH3 domain binding; and the most differentially expressed KEGG protein pathway was dilated cardiomyopathy. PPI analysis revealed PPIs among the top 300 proteins.

**Conclusions:** The SDAVF-DV exhibits specific pathology and protein expression changes under sustained high vascular pressure. The results of the present study provide insights into the pathogenesis of SDAVF formation at the protein level as well as a scientific foundation for further exploration of the pathophysiological mechanism of the SDAVF.

## Introduction

Spinal dural arteriovenous fistula (SDAVF) is the most common spinal vascular shunt lesion characterized by an abnormal connection between a radicular meningeal artery and a radicular medullary vein. As venous connections drain to radicular veins, the draining vein shows gradual arterialization. Because of venous hypertension, clinical presentations and progressive myelopathy can be assessed.

In SDAVF, venous drainage is provided by longitudinal spinal veins linked together and to the epidural network (1, 2). In many clinical case reports, the arterialized SDAVF draining vein (SDAVF-DV) was identified easily after opening the dura during an operation. The pathology of the arterialized SDAVF-DV was mentioned in a previous study. However, the protein changes in this arterialized vein under high intravascular pressure remain enigmatic.

In the present study, we used quantitative proteomics to compare the SDAVF-DV with the superficial temporal artery (STA) and superficial temporal vein (STV) to show different protein expression levels under venous hypertension. The results of our present study might provide insights into the pathogenesis of SDAVF formation at the protein level.

## Methods

### Ethics Statement

The current study was examined and approved by the Ethics Committee of Huashan Hospital, Fudan University. Each participant provided their written informed consent to participate in this study.

### Patients and Tissue Sample Preparation

Three SDAVF-DVs were removed after microsurgery ligation. Three STAs and three STVs were obtained from patients with intracranial tumors via the extended pterional approach (3, 4). We used the samples from each group for the comparative proteomics analysis. The tissues used for the proteomics analysis were immediately frozen in liquid nitrogen and stored at −80°C. The SDAVF-DVs, STAs, and STVs were homogenized in a 4% sodium dodecyl sulfate (SDS), 100 mM of Tris-HCl, and 100 mM of DTT solution. Then, a fluorescence assay was conducted to determine the total protein concentration. Approximately 200 μg of total protein from the tissues was proteolysed on a 10-kDa filter (PALL Life Sciences, Shanghai, China) using a Filter Aided Sample Preparation (FASP) protocol as described in detail elsewhere (5). The peptide solution was transferred to a solid-phase extraction cartridge (Empore 7 mm/3 ml) for desalting and cleanup. The peptide samples were resuspended in water with 0.1% formic acid (v/v), and the protein content was estimated by UV light spectral density at 280 nm (6) prior to analysis by nano-liquid chromatography–tandem mass spectrometry (N-LC-MS/MS).

### H and E Staining, Masson Staining, and Immunostaining

Sections were randomly chosen from each sample for hematoxylin and eosin staining (H&E), Masson's trichrome staining (Sigma, St. Louis, MO, USA), and immunohistochemistry. Anti-von Willebrand factor (vWF) (Abcam, Cambridge, MA, USA), anti-α-smooth muscle actin (α-SMA) (Invitrogen, Carlsbad, CA, USA), anti-angiotensin receptor 1 (AT1) (Abcam), anti-piezo-1 (Novus Biologicals, Cambridge, UK), anti-elastin (Abcam), anti-collagen I (COL I) (Abcam), anti-collagen III (COL III) (Abcam), anti-collagen IV (COL IV) (Abcam), anti-collagen VI (COL VI) (Abcam), anti-CD45 (Abcam), anti-CD68 (Abcam), anti-COX-1 (Cell Signaling Technology, Danvers, MA, USA), and anti-COX-2 (Abcam) were used as the primary antibodies. 3,3′-Diaminobenzidine (DAB) plus chromogen (Thermo Fisher Scientific, Waltham, MA, USA) was used for substrate visualization according to the manufacturer's protocol.

### Label-Free Quantitative Analysis and Data Processing

Trypsin-digested peptides from the tissues were analyzed by LC-MS/MS; each sample was analyzed twice. All raw Xcalibur files acquired from the MS runs were analyzed using the default settings of MaxQuant software (version 1.3.0.5) with minor modifications as previously described (7). Hierarchical clustering was performed with MEV software (v4.6, TIGR). The differentially expressed proteins (*p* < 0.05) were analyzed by hierarchical clustering to identify potential markers capable of classifying all samples.

The clustering pattern, expression analyses, and volcano plots were based on R software according to standardized data. Venn diagrams of the characteristics of each of the three groups of differentially expressed proteins were generated.

The Gene Ontology (GO) and enrichment analyses of the dysregulated proteins in this experiment were based on the publicly available databases DAVID 6.7 (http://david.abcc.ncifcrf.gov/) and QuickGO (http://www.ebi.ac.uk/QuickGO/).

The genomic, chemical, and systemic functions of the dysregulated proteins were analyzed and enriched by Kyoto Encyclopedia of Genes and Genomes (KEGG) analysis (http://www.kegg.jp/kegg/pathway.html). The significance of differential protein enrichment in each pathway entry was calculated using the hypergeometric distribution test and is expressed as the *p*-value. Predicted protein–protein interaction (PPI) networks for these differentially expressed proteins were constructed using the STRING database (http://string.embl.de/) and Cytoscape software (http://www.cytoscape.org/).

### Statistical Analysis

The statistical analysis was performed with IBM SPSS, and the graphs were generated with GraphPad Prism software. The significance of differences between two groups in the proteomics analysis was assessed using one-way analysis of variance (ANOVA). Proteins were defined as significantly differentially expressed when the ratio was ≥2 or ≤0.5 in the SDAVF-DV compared with normal tissues (*p* < 0.01).

## Results

### Differences in Pathology Among the Spinal Dural Arteriovenous Fistula Draining Vein, Superficial Temporal Artery, and Superficial Temporal Vein

**Figure 1:**
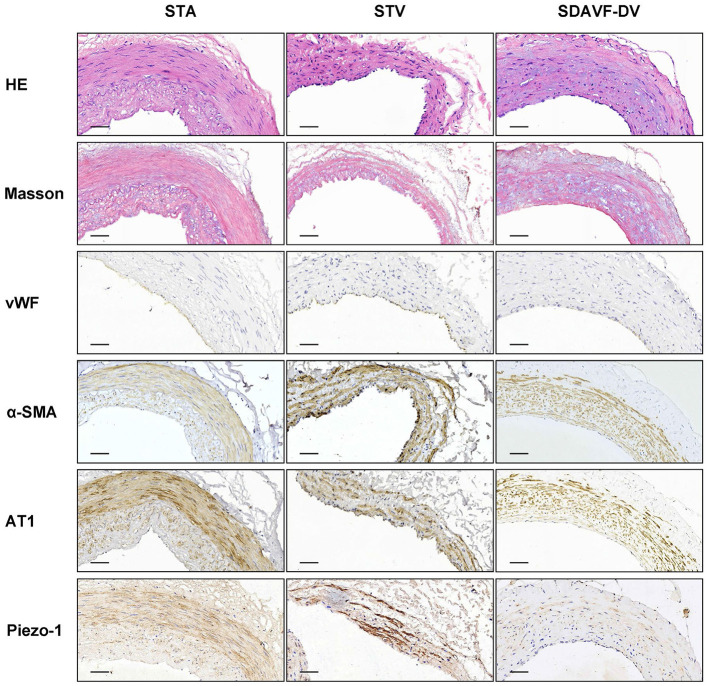
HE staining, Masson's trichrome staining, vWF, α-SMA, AT1 and piezo-1 immunohistochemistry. Histological features of the STA, STV and SDAVF-DV are shown in the photomicrographs. Representative photomicrographs showing HE-stained and Masson's trichrome-stained vascular tissues are presented. vWF, α-SMA, AT1 and piezo-1 were detected by immunohistochemistry. Scale bar = 50 μm in immunohistochemistry.

**Figure 2:**
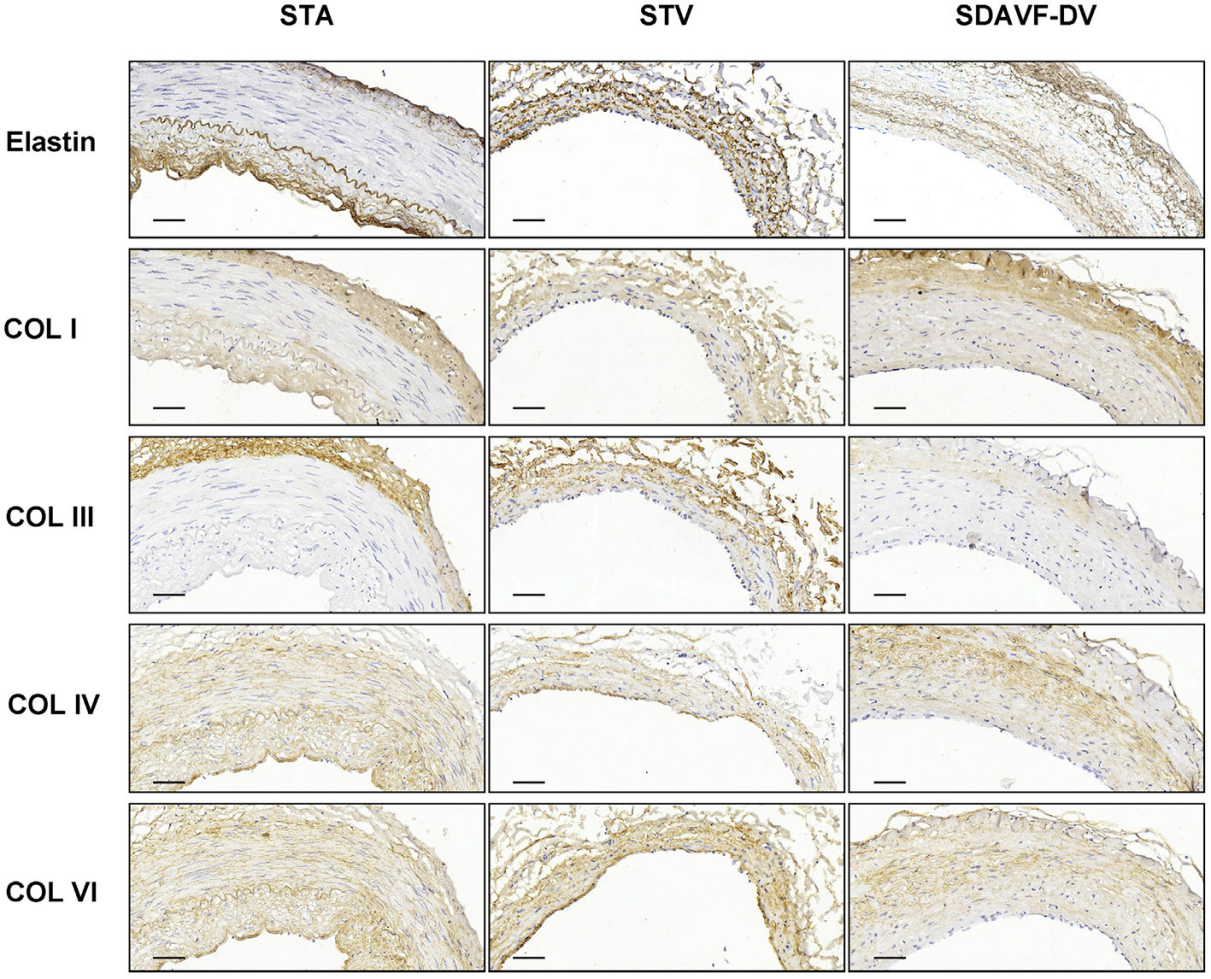
Elastin, COL I, COL III, COL IV and COL IV immunohistochemistry. Histological features of the STA, STV and SDAVF-DV are shown in the photomicrographs. Representative photomicrographs showing HE-stained and Masson's trichrome-stained vascular tissues are presented. Elastin, COL I, COL III, COL IV and COL IV were detected by immunohistochemistry. Scale bar = 50 μm.

**Figure 3:**
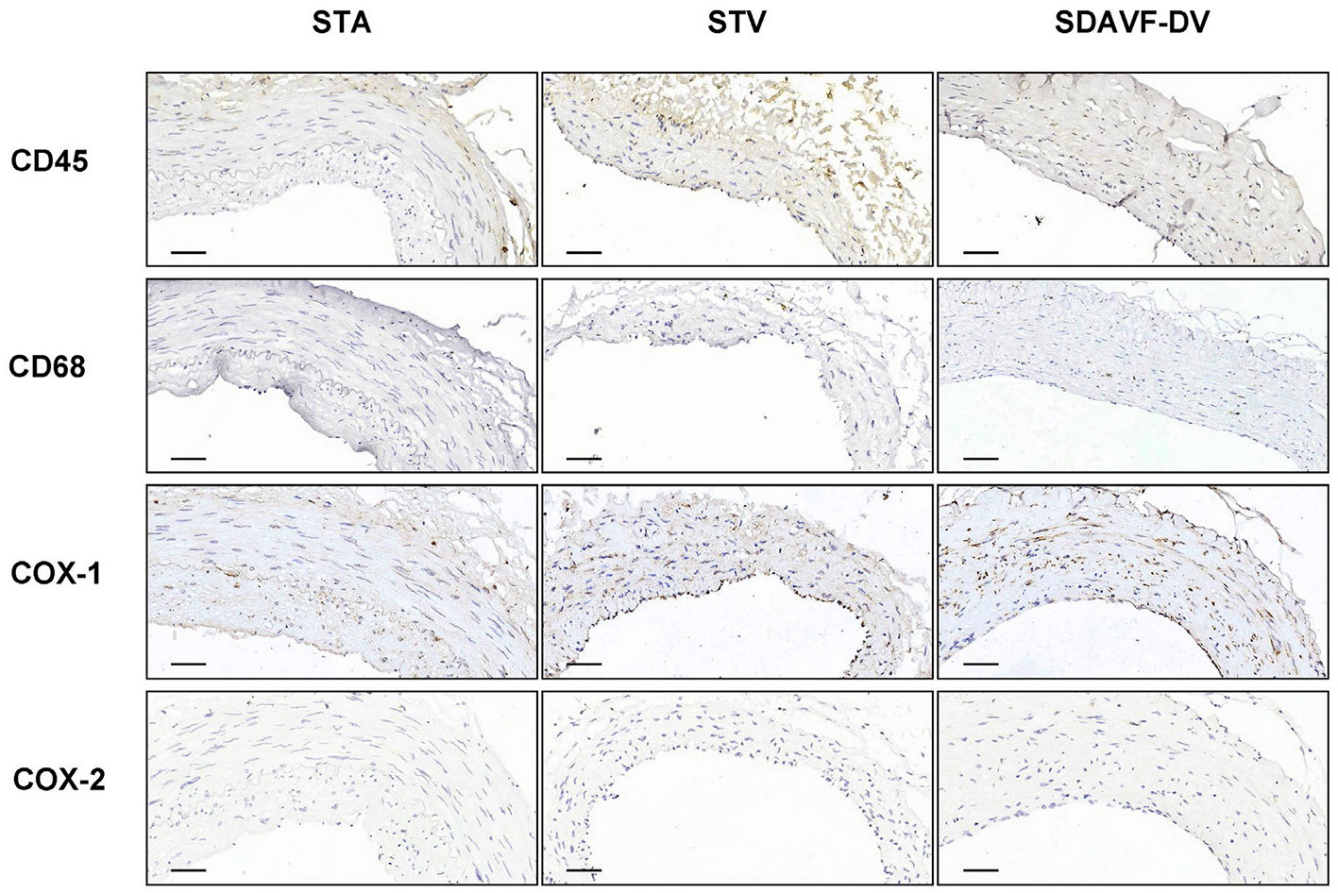
CD45, CD68, COX-1 and COX-2 immunohistochemistry. Histological features of the STA, STV and SDAVF-DV are shown in the photomicrographs. Representative photomicrographs showing HE-stained and Masson's trichrome-stained vascular tissues are presented. CD45, CD68, COX-1 and COX-2 were detected by immunohistochemistry. Scale bar = 50 μm in immunohistochemistry.

H&E and Masson staining showed an artery-like structure of the SDAVF-DV. Immunostaining revealed that vWF+ cells were not continuous. α-SMA+ and AT1+ cells showed an increase in smooth muscle cells, and the cell distribution was disordered in the SDAVF-DV. Piezo-1 expression was lower in the SDAVF-DV tunica media (Figure 1). The distributions of elastin, COL I, and COL III was different in the SDAVF-DV. COL IV and COL VI were decreased in the SDAVF-DV (Figure 2). The SDAVF-DV exhibited more CD45+ cells and COX-1 expression than did the controls. No differences in CD68 expression and COX-2 staining were observed between the SDAVF-DV and controls (Figure 3).

### Identification of Differentially Expressed Proteins in the Spinal Dural Arteriovenous Fistula Draining Veins, Superficial Temporal Arteries, and Superficial Temporal Veins

Three paired SDAVF-DV, STA, and STV tissue samples were analyzed in the initial discovery phase.

**Figure 4:**
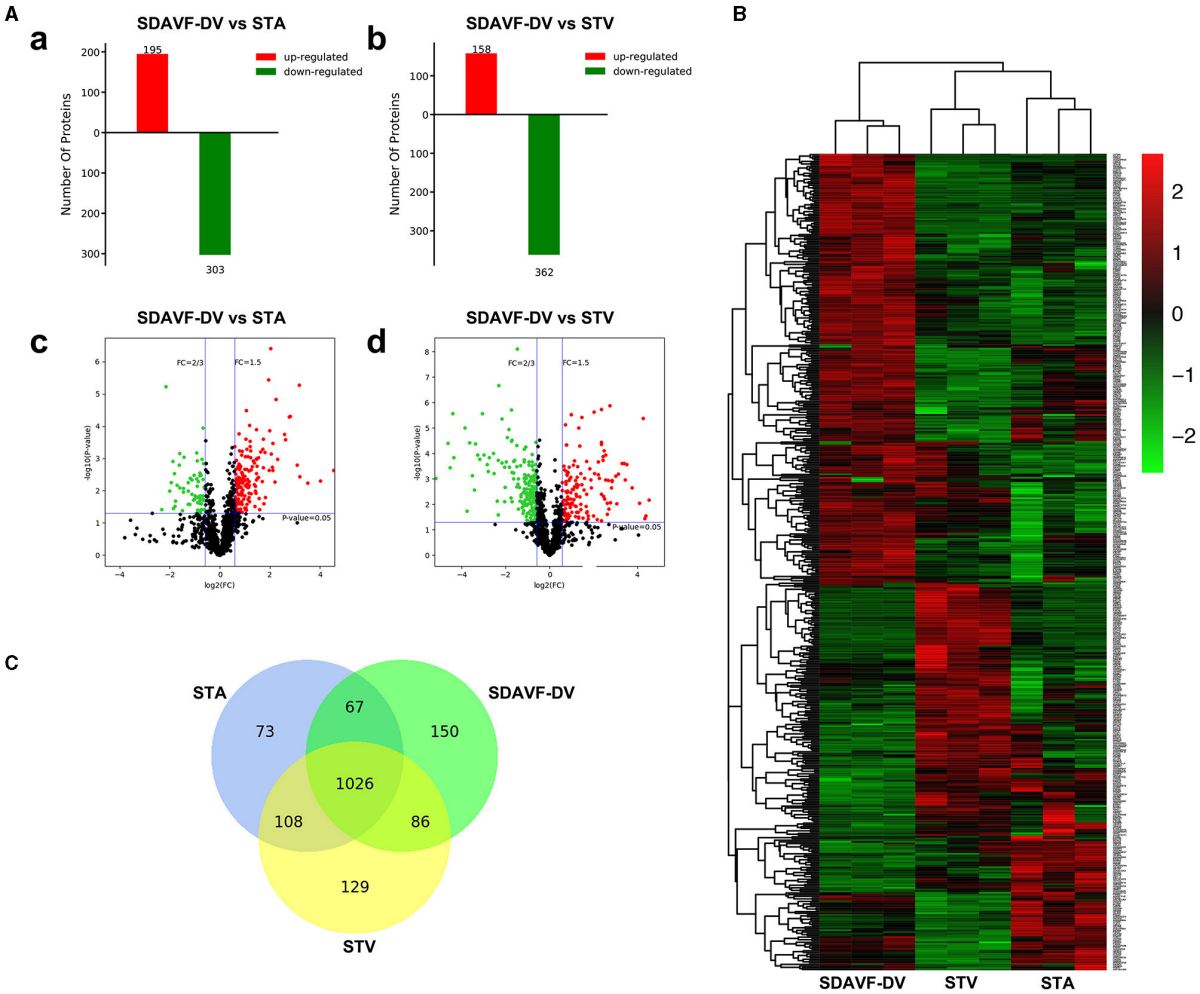
Differentially expressed proteins identified by label-free quantitative proteomics. **(A)** Significant differences in protein levels in the SDAVF-DV compared with the STA (a) and STV (b). Volcano plot showing protein changes in the SDAVF-DV compared with the STA (c) and STV (d). **(B)** Heatmap of 480 proteins expressed in the SDAVF-DV, STA and STV. **(C)** Venn diagram showing overlap of protein expression.

An equal amount of protein from each tissue was digested. Then, the peptides were analyzed by N-LC-MS/MS. Using MaxQuant (version 1.3.0.5), we identified 2,829 nonredundant proteins with a local false discovery rate (FDR) <1% and at least two unique peptides per protein. The label-free quantification (LFQ) intensity ratios for the 2,829 proteins were calculated, and significant differences in the protein expression levels between two tissues were determined using a *t*-test (*p* < 0.05). Compared with the STA, the SDAVF-DV had 195 significantly upregulated proteins and 303 significantly downregulated proteins. Compared with the STV, among the 520 proteins that exhibited significant differences, 158 were significantly upregulated and 362 were significantly downregulated in the SDAVF-DV (Figure 4A). When the three groups were combined, 480 differentially expressed proteins were identified (shown in the heatmap in Figure 4B). Venn analysis showed the variation and commonalities of different proteins in each group. A total of 1,026 proteins were expressed in all groups, and 150 proteins were identified only in the SDAVF-DV (Figure 4C).

### Gene Ontology Analysis

We performed GO analysis to analyze the differentially expressed proteins. When comparing the SDAVF-DV with the STA, most of differential GO terms expressed in each category were the adenylate cyclase-modulating G protein-coupled receptor signaling pathway, U6 snRNP, and SH3 domain binding. We examined the top 10 upregulated and downregulated GO terms in the biological process, cellular component, and molecular function categories with 2.0-fold (*p* < 0.05) differential gene expression (Table 1).

**Table 1:** Top 10 GO term (SDAVF-DV vs STA).

**Category**	**Top 10 upregulated GO term**	**Top 10 downregulated GO term**
Biological process		
	adenylate cyclase-modulating G-protein coupled receptor signaling pathway	translation reinitiation
	lamellipodium assembly	IRES-dependent translational initiation
	lamellipodium morphogenesis	ribosome disassembly
	regulation of epithelial cell migration	regulation of growth
	regulation of microtubule-based process	regulation of cardiac muscle hypertrophy
	wound healing	positive regulation of calcineurin-NFAT signaling cascade
	regulation of receptor recycling	plasma membrane repair
	ESCRT III complex disassembly	regulation of vascular endothelial growth factor receptor signaling pathway
	regulation of osteoclast differentiation	prostaglandin biosynthetic process
	trophectodermal cell differentiation	secretion
Cellular component		
	nuclear pore outer ring	U6 snRNP
	GATOR2 complex	Lsm1-7-Pat1 complex
	mitochondrial envelope	ESCRT III complex
	elastic fiber	cytoplasmic side of plasma membrane
	GAIT complex	membrane coat
	intermediate filament	endoplasmic reticulum exit site
	neurofilament cytoskeleton	nuclear outer membrane
	microfibril	rough endoplasmic reticulum
	membrane raft	LINC complex
	late endosome membrane	smooth muscle contractile fiber
Molecular function		
	SH3 domain binding	phospholipid binding
	antioxidant activity	protein disulfide oxidoreductase activity
	ATP-dependent NAD(P)H-hydrate dehydratase activity	lyase activity
	ADP-dependent NAD(P)H-hydrate dehydratase activity	glutathione binding
	phosphatidylinositol phospholipase C activity	prostaglandin-E synthase activity
	phospholipase C activity	catalytic activity
	metallopeptidase activity	protein serine/threonine phosphatase inhibitor activity
	G-protein coupled serotonin receptor binding	peroxidase activity
	3-hydroxyacyl-CoA dehydrogenase activity	interleukin-1 receptor antagonist activity
	acetyl-CoA C-acyltransferase activity	proteinase activated receptor binding

When comparing the SDAVF-DV with the STV, and the most differential GO terms expressed in each category were lamellipodium assembly, U6 snRNP, and SH3 domain binding.

**Figure 5:**
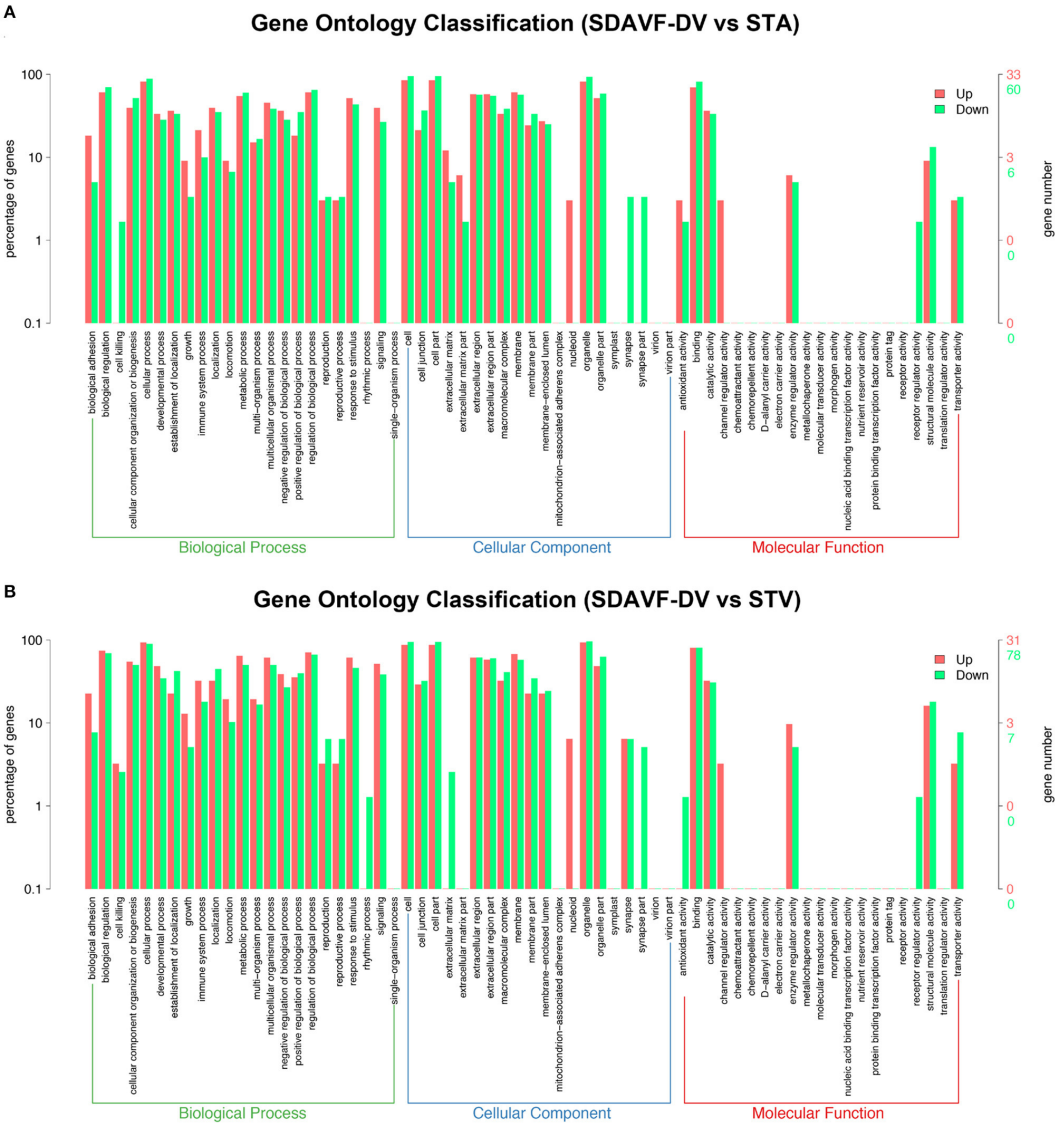
GO analysis of significantly dysregulated proteins and changes in the SDAVF-DV. GO analysis of the up- and downregulated GO terms for the 2.0-fold differentially expressed genes associated with the biological process, cellular component and molecular function categories when comparing the SDAVF-DV with the STA **(A)** and STV **(B)**.

The top 10 upregulated and downregulated GO terms based on the comparison of the SDAVF-DV and STV are also listed in Table 2. In GO classification, 93 differentially enriched GO terms were found between the SDAVF-DV and STA: 60 GO terms were upregulated and 33 were downregulated (Figure 5A). Compared with the STV, the SDAVF-DV had 109 differentially enriched GO terms: 31 terms were upregulated and 78 were downregulated (Figure 5B).

**Table 2:** Top 10 GO term (SDAVF-DV vs STV).

**Category**	**Top 10 upregulated GO term**	**Top 10 downregulated GO term**
Biological process		
	lamellipodium assembly	exonucleolytic nuclear-transcribed mRNA catabolic process involved in deadenylation-dependent decay
	lamellipodium morphogenesis	ubiquitin homeostasis
	neuromuscular synaptic transmission	endosomal vesicle fusion
	dGTP catabolic process	positive regulation of pinocytosis
	regulation of innate immune response	regulation of cardiac muscle hypertrophy
	dATP catabolic process	positive regulation of calcineurin-NFAT signaling cascade
	regulation of receptor recycling	early endosome to Golgi transport
	ESCRT III complex disassembly	regulation of cardiac muscle contraction
	positive regulation of epithelial to mesenchymal transition	protein targeting to plasma membrane
	positive regulation of glycogen biosynthetic process	positive regulation of voltage-gated calcium channel activity
Cellular component		
	zonula adherens	U6 snRNP
	cell-substrate adherens junction	Lsm1-7-Pat1 complex
	sarcoplasm	Cajal body
	mitochondrial small ribosomal subunit	macropinosome
	mitochondrial oxoglutarate dehydrogenase complex	ciliary pocket membrane
	mitochondrial envelope	ESCRT III complex
	phosphopyruvate hydratase complex	cytoplasmic side of plasma membrane
	alphav-beta3 integrin vitronectin complex	membrane coat
	cytosolic ribosome	smooth muscle contractile fiber
	microtubule minus-end	integrin alpha8-beta1 complex
Molecular function		
	SH3 domain binding	phospholipase A2 activator activity
	dGTPase activity	PDZ domain binding
	dGTP binding	phosphate ion binding
	insulin receptor binding	phosphatidylinositol phosphate binding
	3-hydroxyacyl-CoA dehydrogenase activity	phospholipid binding
	acetyl-CoA C-acyltransferase activity	cysteine-type peptidase activity
	enoyl-CoA hydratase activity	catalytic activity
	long-chain-3-hydroxyacyl-CoA dehydrogenase activity	peroxidase activity
	3-hydroxyacyl-CoA dehydrogenase activity	interleukin-1 receptor antagonist activity
	acetyl-CoA C-acyltransferase activity	muscle alpha-actinin binding

### Kyoto Encyclopedia of Genes and Genomes Pathway Analysis

**Figure 6:**
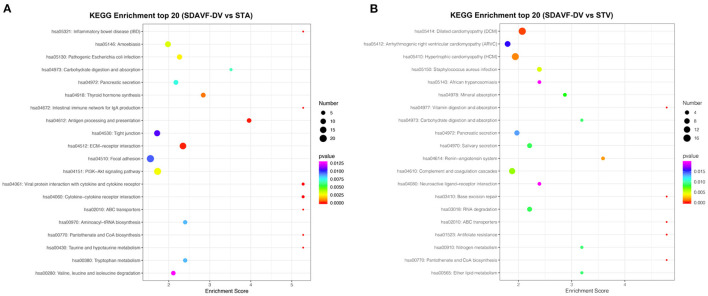
KEGG pathway analysis. The bubble chart shows the KEGG pathway analysis of the top 20 differentially expressed genes when comparing the SDAVF-DV with the STA **(A)** and STV **(B)**.

The KEGG pathway analysis of these differentially expressed proteins also demonstrated related pathways. Figure 6 shows the number of proteins in each KEGG pathway and the *p*-value of the top 20 pathways. Compared with the STA, the top three differentially expressed protein pathways were focal adhesion, the PI3K-Akt signaling pathway, and the extracellular matrix (ECM)–receptor interaction. Compared with the STV, the top three differentially expressed pathways were dilated cardiomyopathy, hypertrophic cardiomyopathy, and adrenergic signaling in cardiomyocytes.

### Protein-Protein Interaction Analysis

**Figure 7:**
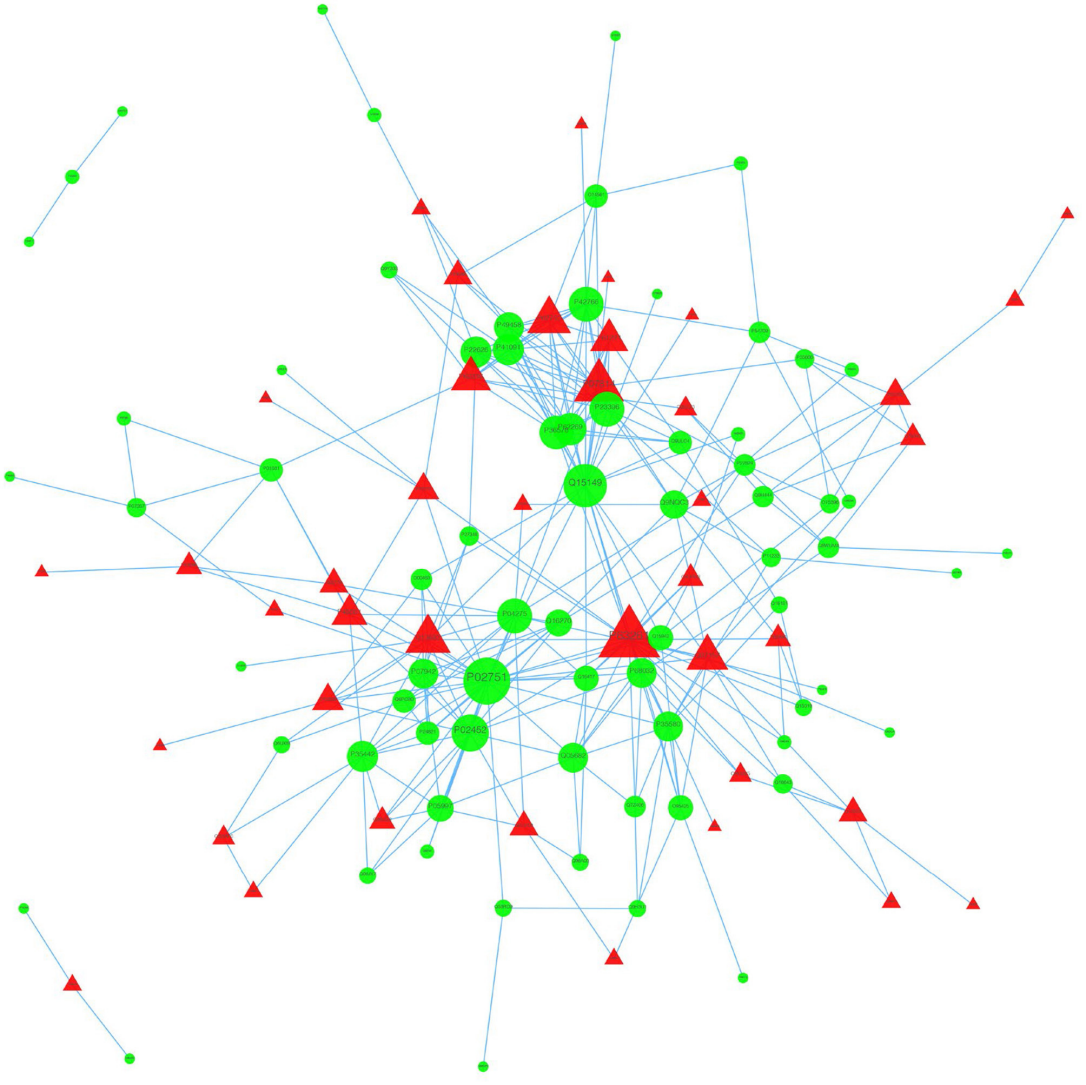
Visualization of PPIs between the SDAVF-DV and STA. Visualization of PPIs for the top 300 proteins using STRING analysis. Red represents upregulated proteins, and green represents downregulated proteins.

**Figure 8:**
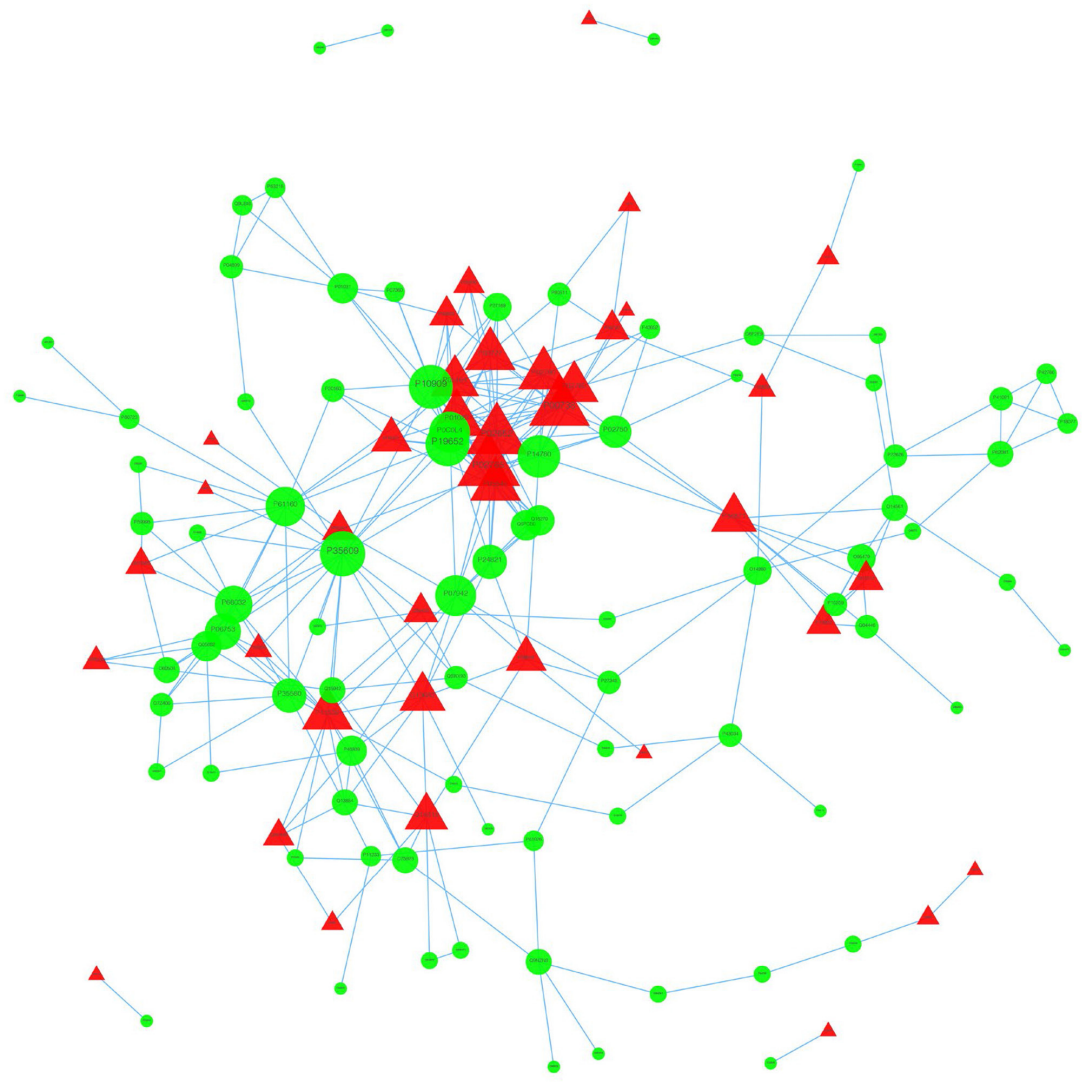
Visualization of PPIs between the SDAVF-DV and STV. Visualization of PPIs for the top 300 proteins using STRING analysis. Red represents upregulated proteins, and green represents downregulated proteins.

We used the STRING database to analyze the differentially expressed proteins, obtain the interactions/relationships among the differentially expressed proteins, and calculate the combined score. We selected the top 300 proteins and found significant PPIs among them (Figures 7, 8). Compared with the STA and STV, the SDAVF-DV showed upregulated and downregulated proteins, and the top three interaction proteins are listed in Table 3.

**Table 3:** Top three interaction protein number proteins.

	**Gene ID**	**Upregulated protein**
SDAVF-DV vs. STA		
	P63261	Actin, cytoplasmic 2
Upregulated	P07814	Bifunctional glutamate/proline–tRNA ligase
	P62750	60S ribosomal protein L23a
	P02751	Fibronectin
Downregulated	Q15149	Plectin
	P02452	Collagen alpha-1(I) chain
SDAVF-DV vs STV		
	P00738	Haptoglobin
Upregulated	P02652	Apolipoprotein A-II
	P02765	Alpha-2-HS-glycoprotein
	P19652	Alpha-1-acid glycoprotein 2
Downregulated	P10909	Clusterin
	P14780	Matrix metalloproteinase-9

## Discussion

SDAVF is a common arteriovenous shunt located inside the dura mater close to the spinal nerve root (8). Venous hypertension, which induces medullar venous outflow disturbances, results in chronic hypoxia and congestive myelopathy (9). The direct intraoperative measurement of the vascular pressure in the fistula can be as high as 74% of the systemic arterial pressure (10, 11). This finding may explain why, in some patients, symptoms become worse during physical activity with a concomitant increase in arterial pressure (12, 13). Most previous studies have focused on the histology and anatomy of the SDAVF-DV. Based on intraoperative findings, we demonstrated protein changes in the arterial SDAVF-DV.

Previous studies had revealed that many angiogenetic factors including vascular endothelial growth factor (VEGF), platelet-derived growth factor (PDGF), transforming growth factor (TGF), RAS-MAPK-ERK signal pathway, and PI3K/Akt signal pathway could promote cerebral vascular malformations formation (14). Besides, hyperactivation of oncogenic KRAS in endothelial cells could induce sporadic brain arteriovenous malformations *in vivo* (15, 16). Under long-period venous hypertension, draining vein arterialization begins. There is a growing body of evidence to suggest that angiogenesis plays a role in SDAVF development. The high expression of angiogenic growth factors (especially VEGF) had been proven in human DAVF specimens (17, 18). Once sinus thrombosis and venous hypertension are initiated, hypoxia-inducible factor 1 (HIF-1), which was known to be an important upstream regulator of VEGF, was released very rapidly (19). And animal experiments also indicated that release of VEGF was highly related with DAVF formation and that VEGF inhibitor could inhibit its development (20, 21). Some genetic changes were proved within arterialized veins. The surgical method enabled drainage vein to be preserved, and molecular alterations were found in the arterialization of a vein (22).

Pathology analysis revealed thickening of the vascular wall and hyperplasia of the smooth muscle cell layers in the SDAVF-DV compared with the STV. In the tunica media, most cells expressed α-SMA and AT1, but piezo-1-positive cells were decreased in the SDAVF-DV, suggesting disorder of piezo-1 mechanosensitive channels in smooth muscle cells of the SDAVF-DV. The distribution of the ECM showed a special vascular structure under sustained high vascular pressure.

A clear understanding of the mechanism of SDAVF development is still lacking. Our study was the first to perform a comparative proteome analysis and show the differential expression of proteins in arterialized SDAVF-DVs compared with normal arteries and veins. In general, most of the proteins were the same between the three groups. Because of its special pathophysiology, the SDAVF-DV showed specific protein expression compared with the STA and STV.

In the intraoperative observation, the SDAVF-DV showed arterial morphology. Thron proposed a hypothesis based on spine arteriovenous shunt anatomy (13). Takai showed that the vessel wall of the proximal subarachnoid portion of the intradural draining vessels was irregularly thickened by collagen, exhibited elastic fibrosis, and was without a continuous internal elastic lamina and a regular smooth muscle layer. The diameter of the vessels was significantly enlarged (23).

After GO analysis, the SDAVF-DV showed a decrease in smooth muscle contractile fibers, which might indicate smooth muscle cell dysfunction. This might be induced by long-range venous hypertension stretching on the SDAVF-DV. Stretch plays an important role in maintaining smooth muscle cell function and regulating inflammation. A former study showed that mechanical stretch-induced endoplasmic reticulum stress, apoptosis, and inflammation contribute to thoracic aortic aneurysm and dissection (24). In our research, we also identified that mechanical stretching could induce smooth muscle cell changes from a contract phenotype to an inflammatory phenotype (25, 26). The regulation of inflammatory factors is related to the hypothesis on SDAVF formation. In THE KEGG and PPI analyses, the ECM and focal adhesion showed obvious changes. Degeneration of the ECM is primarily induced by the secretion of inflammatory cytokines and cell infiltration in cerebral vascular disease (27–29). During SDAVF formation, inner vessel wall inflammation might contribute to an insufficient ECM and trigger changes in pathological proteins.

## Conclusions

To our knowledge, few studies have focused on the SDAVF-DV. However, several researchers have investigated its pathological characteristics (23, 29). We first examined protein changes to determine whether the lesion vessel was an artery, vein, or vein-to-artery transition.

In this study, we found the vessel wall of SDAVF-DV remodeling, which is different with artery and vein histologically even it is similar to artery in morphology. The results of proteomics showed smooth muscle cell dysfunction and inflammation changes of SDAVF-DV. Our study adds new information on the formation of SDAVF to the realm of protein changes in the draining vein using proteomics. This finding may shed light on the mechanism of SDAVF formation.

## Data Availability Statement

The raw data supporting the conclusions of this article will be made available by the authors, without undue reservation.

## Ethics Statement

The studies involving human participants were reviewed and approved by Ethics Committee of Huashan Hospital, Fudan University. The patients/participants provided their written informed consent to participate in this study.

## Author Contributions

PL: conception and design of study, provision of study materials, collection and/or assembly of data, data analysis and interpretation, manuscript writing, and final approval of manuscript. YS: data analysis and interpretation, and manuscript writing. SL and YL: database input and data interpretation. YZ: data analysis. YS: manuscript writing. WZ: provision of study and revision and final approval of manuscript. QA: conception and design of study and revision and final approval of manuscript. WZ and QA: are co-corresponding authors of this article. All authors contributed to the article and approved the submitted version.

## Funding

The Outstanding Academic Leaders Program of Shanghai Municipal Commission of Health and Family Planning (No. 2017BR006 to WZ); National Natural Science Foundation of China (No. 81571102, No. 81870911 to WZ; No. 81801148 to PL). This study was supported by Clinical Research Plan of SHDC (No. SHDC2020CR2034B to WZ, No. SHDC2020CR4033 to KQ).

## Conflict of Interest

The authors declare that the research was conducted in the absence of any commercial or financial relationships that could be construed as a potential conflict of interest.

## Publisher's Note

All claims expressed in this article are solely those of the authors and do not necessarily represent those of their affiliated organizations, or those of the publisher, the editors and the reviewers. Any product that may be evaluated in this article, or claim that may be made by its manufacturer, is not guaranteed or endorsed by the publisher.

## Abbreviations

SDAVF: spinal dural arteriovenous fistula
SDAVF-DV: spinal dural arteriovenous fistula draining vein
STA: superficial temporal artery
STV: superficial temporal vein
KEGG: Kyoto Encyclopedia of Genes and Genomes.
